# Development and Application of a TaqMan-Based qPCR Assay for Detecting ENTV-2 in Goats

**DOI:** 10.3390/genes16050529

**Published:** 2025-04-29

**Authors:** Pengfei Li, Haike Yin, Xiaoan Cao, Xi Lan, Jinyan Wu, Jijun He, Ligang Yuan, Youjun Shang

**Affiliations:** 1 State Key Laboratory for Animal Disease Control and Prevention, Lanzhou Veterinary Research Institute, Chinese Academy of Agricultural Sciences, Lanzhou 730046, China; pengfei0957@163.com (P.L.); caoxiaoan@caas.cn (X.C.); lanxi@caas.cn (X.L.); wujinyan@caas.cn (J.W.); hejijun@caas.cn (J.H.); 2 College of Veterinary Medicine, Gansu Agricultural University, Lanzhou 730070, China; 3 Baoji Animal Husbandry and Veterinary Center, Baoji 721001, China; yinhaike2025@163.com

**Keywords:** enzootic nasal tumor virus 2, enzootic nasal adenocarcinoma, *pro* gene, RT-qPCR

## Abstract

Background: In recent years, enzootic nasal tumor virus 2 (ENTV-2) has become prevalent in China, resulting in substantial economic losses for the goat industry. In order to enrich the availability of detection methods for ENTV-2, this study developed an expedited and accurate reverse-transcription quantitative real-time polymerase chain reaction (RT-qPCR) assay to facilitate the detection and quantification of ENTV-2. Methods: Specifically, a pair of primers and a TaqMan probe targeting conserved regions of the *pro* gene were designed to allow the specific amplification and detection of viral RNA in clinical samples. Moreover, modifying the method for use in a quantitative real-time PCR (qPCR) assay enables the detection of proviral DNA in tumor specimens. Results: Both methods exhibited a detection limit for the ENTV-2 standard plasmid at 10^0^ copies/µL. The detection methods we established exhibited high specificity and sensitivity to ENTV-2, without cross-reactivity with other pathogens causing respiratory diseases or endogenous retroviruses (EBRVs). We performed an ENTV-2 analysis of clinical samples in goats via RT-qPCR using nasal swab samples (*n* = 558) collected from three geographically distinct flocks in Lingyou County, Baoji City, Shaanxi Province, China, and 58 positive samples were detected for a positivity rate of 10.4%. After euthanasia, the autopsy report showed nasal cavity masses. Histopathological analysis demonstrated an epithelial neoplasm, in compliance with the features of enzootic nasal adenocarcinoma (ENA). Three full-length genomes were sequenced to assess genomic sequence conservation and variation. Multiple-sequence alignment demonstrated the existence of sequence variations among strains. Phylogenetic analysis of the nucleotide sequences revealed that the ENTV-2 SX1~3 isolates were phylogenetically related to the Chinese ENTV-2 isolates, especially the JY strain. Furthermore, recombination analysis suggested that both ENTV-2 SX1 and ENTV-2 SX2 might be recombinant variants. Conclusions: In conclusion, both methods are highly specific for the *pro* gene of ENTV-2, and the development of this assay has been deemed crucial to the early identification and subsequent control of this viral infection. Our results provide valuable information for further research on the genetic variation and evolution of ENTV-2 in China.

## 1. Background

Enzootic nasal tumor virus 2 (ENTV-2), the etiological agent of enzootic nasal adenocarcinoma (ENA) in goats, mainly causes contagious respiratory diseases in goats, characterized by the neoplastic transformation of secretory epithelial cells in the upper respiratory tract [1]. Thus far, ENA tumors have been classified as low-grade adenocarcinoma, which does not metastasize to other organs [2,3]. ENTV-2 [1] and enzootic nasal tumor virus 1 (ENTV-1) [4] are known to be two phylogenetically distinct virus types which infect goats and sheep, respectively. Both ENTV-2 and ENTV-1 are classified under the genus *Betaretrovirus* of the family *Retroviridae*. Gene sequence analysis has shown similarities among ENTV-2, ENTV-1, and jaagsiekte sheep retrovirus (JSRV) in the *Betaretrovirus* genus [2]. The viral genome is a single-stranded positive-sense RNA about 7.5 kb in length and has high homology to endogenous betaretroviruses (EBRVs) of small ruminants [5].

Clinical signs of ENA may include seromucous nasal discharge, respiratory distress, exophthalmos, and facial deformities, eventually leading to weight loss and death [6]. In the initial stage, clinical signs in affected goats are similar to those of other respiratory diseases. Therefore, pathological examination is an important diagnosis method for ENA. There are currently no vaccines available for this virus, and consequently, disease control relies on regular flock inspections and the prompt culling of suspected cases. There is also no known virus-specific humoral immune response following ENTV-2 infection [7]. Consequently, the serological diagnosis of ENTV-2 infection is hampered. At present, diagnosis depends on clinical and pathological investigations of ENTV-2 in combination with identification of the infectious agent by PCR targeting the ENTV-2-specific gene [8]. In addition, ENTV-2 and EBRV sequences share a high degree of sequence similarity. Therefore, they can interfere with molecular detection assays. During the long incubation period, early clinical signs can be easily confused with those of other respiratory diseases, and it is not possible to distinguish healthy from infected animals. This leads to great difficulties in the diagnosis of this disease [9].

It has been observed that aside from Australia and New Zealand [10], ENA has been detected across various nations, including France, India, Spain, Canada, Italy, Greece, China, Turkey, Saudi Arabia, the USA, Iran [8], Brazil [11], and Egypt [12]. In China, beginning with its initial detection in Inner Mongolia in 1995, it has also been progressively found within goat populations across other provinces such as Hunan, Sichuan, Anhui, Shaanxi, Guizhou, Fujian, Chongqing [13,14], and Guangdong [15]. This trend suggests a notable rise in ENA cases, which can potentially have significant economic implications for China’s goat industry. The current state of research on ENTV-2 is limited and predominantly covers isolated instances, largely due to the lack of a comprehensive diagnostic approach. To be able to investigate the spread of ENTV-2 in nature, where the virus is now endemic, a new detection tool is necessary. In this study, we establish a probe-based RT-qPCR assay for detecting ENTV-2, and clinical samples are tested from three geographically distinct goat flocks in Lingyou County, Baoji City, Shaanxi Province, China using the RT-qPCR assay. In addition, we describe the clinical, gross, histopathological, and molecular findings.

## 2. Materials and Methods

### 2.1. DNA and RNA Extraction

Viral RNA and DNA extraction from samples was performed using a TIANamp genomic DNA/RNA kit (Tiangen Biotech Co., Ltd., Beijing, China) while following the manufacturer’s protocol. All RNA and DNA samples were stored at −80 °C.

### 2.2. Primers and Probe Design

**Figure 1:**
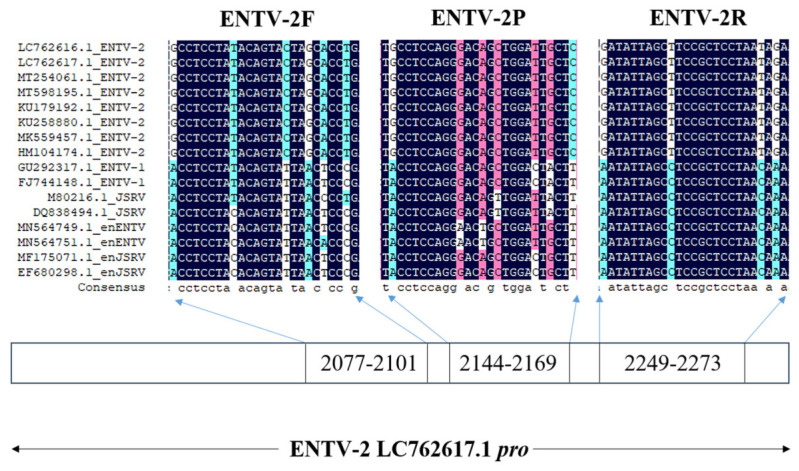
The design of the primers and TaqMan probe for real-time-based methods. Conservation of the corresponding sequence of primers and probe was analyzed using DNAMAN software. The locations of the hybridization sites of the primers and probe for real-time-based methods are indicated on an ENTV-2 (accession number: LC762617.1) isolate *pro* gene.

For the detection of ENTV-2, specific primers (ENTV-2F: GCCTCCTATACAGTACTAGCACCTG; ENTV-2R: GATATTAGCTTCCGCTCCTAATAGA) and a probe (ENTV-2P: TGCCTCCAGGGACAGCTGGATTGCTC) targeting the *pro* gene were designed using Primer Premier software (version 5.0). This process involved retrieving the nucleotide sequences of the *pro* gene from ENTV-2, ENTV-1, jaagsiekte sheep retrovirus (JSRV), and EBRV isolates gathered from the GenBank database. Thereafter, these sequences were aligned using DNAMAN software (version 5.2.2), as depicted in Figure 1. Moreover, the abovementioned probe was labeled with 6-carboxy-fluorescein (FAM) at the 5′-end to enable fluorescence reporting and with Black Hole Quenchers-1 (BHQ-1) at the 3′-end as the quenching dye. To obtain the full-length sequence of ENTV-2, six additional pairs of primers were synthesized, according to the recommendations of a previous study [14]. Furthermore, a BLAST (http://www.ncbi.nlm.nih.gov/tools/primer-blast/) search in the NCBI nucleotide database was performed to confirm the specificity of the designed primers and probe. All primers and the probe were synthesized (Sangon Biotech Co., Ltd., Shanghai, China).

### 2.3. Generation of DNA and RNA Standards

To generate plasmids that contained the target genes, specific cloning primers—forward and reverse—were used to amplify a 197 bp DNA fragment from the DNA template of ENTV-2. This was followed by cloning into a pEASY^®^-T1 cloning vector (Tiangen Biotech Co., Ltd., Beijing, China) and being transformed into *Escherichia coli* DH5a-competent cells (Takara Biomedical Technology Co., Ltd., Beijing, China). The recombinant positive plasmid DNA was extracted using a TIANprep plasmid extraction kit (Tiangen Biotech Co., Ltd., Beijing, China), and it was sequenced by TsingKe (Xian, China). The recombinant plasmids were linearized with HindIII (Takara Biomedical Technology Co., Ltd., Beijing, China) and purified using a TaKaRa MiniBEST DNA Fragment Purification Kit Version 4.0 (Takara Biomedical Technology Co., Ltd., Beijing, China), and the ENTV-2 RNA transcripts were transcribed in vitro using a T7 High Efficiency Transcription Kit (Tiangen Biotech Co., Ltd., Beijing, China) before being treated with DNase I (Tiangen Biotech Co., Ltd., Beijing, China) and purified using an EasyPure^®^ RNA Purification Kit (Tiangen Biotech Co., Ltd., Beijing, China). The concentrations of both the DNA and RNA standards were measured using a spectrophotometer, and then the copy numbers of the recombinant plasmid were calculated as described previously [16,17]. In subsequent experiments, the positive standards were used as standard positive controls and for establishing standard curves for the quantitative analysis.

### 2.4. Development of RT-qPCR and qPCR Methods

Both assays were carried out using Applied Biosystems QuantStudio 5 (Thermo fisher Scientific Inc., Waltham, MA, USA). The RT-qPCR for viral RNA detection was carried out using a One Step PrimeScript^TM^ RT-PCR Kit (Perfect Real Time) (Takara, Dalian, China) with the following reaction system: 2× One-Step RT-PCR Buffer III (10 μL); TaKaRa Ex Taq HS (5 U/μL) (0.4 μL); PrimeScript RT Enzyme Mix II (0.4 μL); each primer (10 μM) (0.4 μL); probe (10 μM) (0.8 μL); and 2 μL of viral RNA and RNase-free dH_2_O to a total volume of 20 μL in each PCR tube. This was then cycled as follows: 42 °C for 5 min, 95 °C for 10 s, and then 45 cycles of 95 °C for 5 s and 60 °C for 20 s.

The qPCR for proviral DNA detection was performed using a Premix Ex Taq^TM^ (Probe qPCR) Kit (Takara, Dalian, China) according to the manufacturer’s instructions. Each PCR reaction included viral DNA (2 µL), Premix Ex Taq (Probe qPCR) (2X) (10 µL), PCR forward primer (10 μM) (0.4 µL), PCR reverse primer (10 μM) (0.4 µL), probe (10 μM) (0.8 µL), and 6.4 μL RNase-free water, up to a total reaction volume of 20 µL. The DNA amplification sequence involved an initial amplification phase at 95 °C for 30 s, followed by a series of 45 cycles fluctuating between 95 °C for 5 s and 60 °C for 30 s.

### 2.5. Specificity and Sensitivity Analysis of ENTV-2 RT-qPCR and qPCR Assay

The specificity of the assay was assessed for other common goat respiratory pathogens. More specifically, RNA or DNA extracted from peste des petits ruminants virus (PPRV), foot-and-mouth disease virus (FMDV), bluetongue virus (BTV), caprine parainfluenza virus type 3 (CPIV3), jaagsiekte sheep retrovirus (JSRV), goatpox virus (GTPV), and orf virus (ORFV) were used as templates for a specificity analysis of ENTV-2 RT-qPCR and the qPCR assay. Meanwhile, RNA and DNA extracted from goat kidney tissue served as the nucleic acid for EBRV, as ENTV-2 is widely distributed in goats, but it is not present in their kidneys [1]. In addition, the *env* gene (accession number: FJ744148.1) was synthesized and subsequently cloned into the pEASY^®^-T1 vector, and named pEASY^®^-T1- ENTV-1- *env*. The positive recombinant plasmids were sequenced by the Sangon Biotech (Shanghai, China) to verify the correctness of the sequence, and the RNA transcripts were synthesized in vitro using a T7 High Efficiency Transcription Kit (TransGen Biotech, Beijing, China), which were then used as an ENTV-1 RNA and DNA template. RNA or DNA isolated from the nasal fluids of healthy goats was used as a negative control (NC).

For the sensitivity analysis of ENTV-2 real-time PCR assay, 10-fold serial dilutions of both RNA and DNA standards were used as templates (range: 10^8^–10^0^ copies/μL) to determine the lowest detection threshold of RT-qPCR and qPCR, and conventional PCR assays were performed for comparison. Each experiment was repeated four times, and regression analysis was performed using an Applied Biosystems QuantStudio 5 (Thermo fisher Scientific, USA) to determine detection limits. PCR products of conventional PCR were analyzed via electrophoresis in 2% agarose gel.

### 2.6. Analysis of Clinical Samples Using the ENTV-2 RT-qPCR Assay

ENTV-2 infection was surveyed in the three geographically distinct goat flocks in Lingyou County, Baoji City, Shaanxi Province, China using the RT-qPCR assay. In total, 558 nasal swab samples were collected from goats. The tumor samples were derived from three goats from three different flocks, all of whom exhibited clinical signs of ENA. Simultaneously, lymph node, trachea, heart, liver, spleen, lung, kidney, and blood samples were collected from the dissected goats for subsequent research. Tumor-like tissues were fixed in 10% buffered formalin for hematoxylin and eosin (H&E) staining as described previously [18]. Histopathological changes were analyzed with a Pannoramic 250 digital slide scanner (3DHISTECH KFT, Budapest, Hungary) [19]. Organelle-level changes in tumors were examined via transmission electron microscopy JEM-1400FLASH (Japan Electron Optics Laboratory Co., Ltd., Tokyo, Japan) according to previously published methods [18,20]. The samples were collected and preserved in a freezer at −80 °C until further use.

### 2.7. Whole-Genome Sequencing and Genomic Analysis

Due to technical limitations of long-range PCR, in order obtain the full-length sequence of ENTV-2, viral RNA extracted from the tumors was used as a template for reverse-transcription PCR (RT-PCR) to amplify 6 overlapping segments. The 6 PCR products were purified (MiniBEST agarose gel DNA extraction kit v.4.0; TaKaRa). Subsequently, the recovered PCR products were cloned into a pMD-18T vector (Takara, China), and 1 independent clone of each of the 6 positive recombinant plasmids was used for DNA sequencing (Sangon Biotech). The full-length genome was assembled using sequence analysis software (Lasergene v.7.1; DNAstar, DNASTAR Inc., Madison, WI, USA). Phylogenetic analyses of ENTV-2 based on the complete genome sequences of the *gag* and *env* genes were performed by the neighbor-joining method using MEGA v7.1 software with 1000 bootstrap replicates. In total, 31 complete sequences of Chinese isolates of ENTV-2 from the GenBank database were compared with ENTV-2 SX1~3 in this study using MEGA v7.1. Then, the potential recombination events were analyzed using RDP software (version v.4.10), and the default parameters were applied. A Bonferroni-corrected threshold of *p* = 0.05 was set. SimPlot software (version 3.5.1) was used to identify the exact breakpoints with BootScan, which performed the bootscan analysis with a sliding window of 200 bp, a step size of 20 bp, a gap strip, and the Kimura 2-parameter substitution model [21].

## 3. Results

### 3.1. The High Specificity, Sensitivity, Repeatability, and Practicability of ENTV-2 RT-qPCR and qPCR Methods

**Figure 2:**
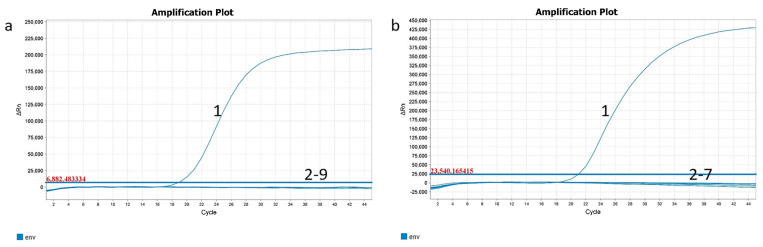
The specificity and analysis of the ENTV-2 real-time PCR assay. (**a**) Specificity for ENTV-2 viral RNA of the RT-qPCR assay. Line 1 = ENTV-2; lines 2–9 = JSRV, ENTV-1, PPRV, FMDV, BTV, CPIV3, EBRV, and negative control, respectively. (**b**) Specificity for ENTV-2 proviral DNA of the qPCR assay. Line 1 = ENTV-2; lines 2–7 = JSRV, ENTV-1, GTPV, ORFV, and EBRV, and negative control, respectively.

Regarding the specificity of the ENTV-2 RT-qPCR assay, only the ENTV-2 viral RNA exhibited a fluorescent signal, while JSRV, ENTV-1, PPRV, FMDV, BTV, CPIV3, and the EBRVs tested negative. The NCs also tested negative (Figure 2a). The developed qPCR assay showed similar results regarding analytical specificity. All of the DNA samples from JSRV, ENTV-1, GTPV and ORFV, as well as from the goat kidney tissue, were negative. The NCs also tested negative (Figure 2b), indicating its high specificity.

Furthermore, using a number of products from the RT-qPCR- and qPCR-positive samples, viral RNA/DNA extracts from the nasal swabs and tumors of the 12 goats (taken from seven herds, collected, and stored in our laboratory) with naturally occurring ENA were sequenced in order to confirm the correct amplification. The sequence analysis showed that the product corresponded to the expected genomic region of the ENTV-2 *pro* gene implying high specificity for the intended target virus ENTV-2.

**Figure 3:**
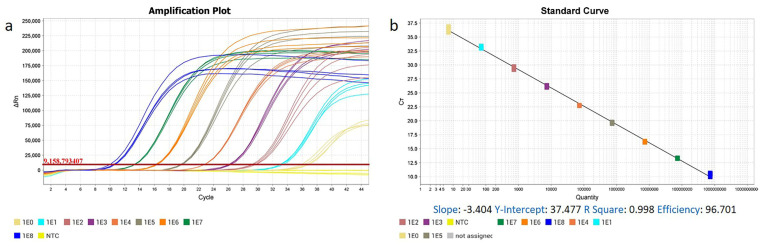

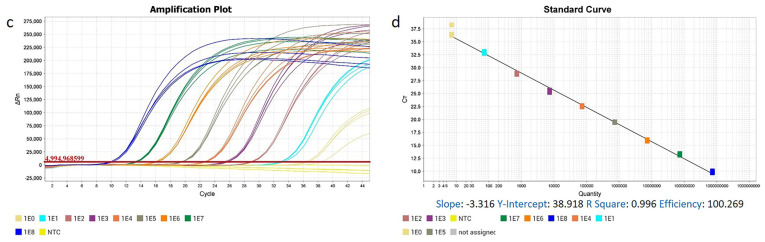
Sensitivity of the real-time PCR for ENTV-2. (**a**) Fluorescence development over time using a dilution range of 10^8^–10^0^ copies of the RNA standard. (**b**) Standard curves of detection of ENTV-2 in the RT-qPCR assay. (**c**) Fluorescence development over time using a dilution range of 10^8^–10^0^ copies of the DNA standard. (**d**) Standard curves of detection of ENTV-2 in the qPCR assay.

**Figure 4:**
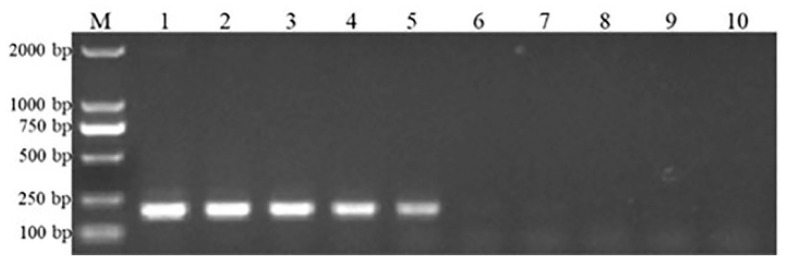
The detection limit of conventional PCR. The sample sequence is consistent with real-time PCR. Lane M = DL2000 DNA marker; lanes 1–9 = the RT-PCR results of plasmid standard with concentrations from 10^8^ copies/μL to 10^0^ copies/μL; lane 10 = negative control.

We also confirmed the sensitivity levels of our established methods. Standard curves for ENTV-2 RT-qPCR (Figure 3a,b) and qPCR (Figure 3c,d) were established using 10-fold serial dilutions of RNA and DNA standards as templates, with each dilution tested in quadruplicate. The coefficient of determination for the RT-qPCR showed good linearity, with R^2^ = 0.998 and slope = −3.404. The amplification efficiency of the RT-qPCR assay was 96.701%, indicating the accuracy of the results and an approximate doubling of the amplicons at each cycle. For qPCR, the standard curve displayed a linear relationship between the Ct value and the copy number, with an R^2^ value of 0.996 and slope of −3.316, while the reaction efficiency was 100.269%. The results of the agarose gel electrophoresis show that the conventional PCR was only able to detect a positive template at concentrations as low as 10^4^ copies/μL (Figure 4). By contrast, both the RT-qPCR and qPCR were able to detect lower concentrations of templates, with the lowest concentration of the standard plasmid detected by the two assays being 10^0^ copies/μL (Figure 3a,c). In comparison with the conventional PCR, the sensitivity of the TaqMan assay was 1000-fold greater (Figure 4). The repeatability of this established assay was evaluated using *pro* region standard RNA and DNA of ENTV-2, with coefficients of variation ranging from 0.098 to 3.740, indicating consistently high reproducibility (Table 1).

### 3.2. Detection and Analysis of Clinical Samples

To further evaluate and apply the methods we established, nasal swab samples (*n* = 558) were collected from three geographically distinct goat flocks in Lingyou County, Baoji City, Shaanxi Province, China, all of which were reared under a combination of semi-captive and semi-grazing methods. Clinical symptoms of ENA were observed in three two-year-old ewes on three different farms characterized by continuous nasal fluid, respiratory distress, and even exophthalmos and skull deformations. No other animals presented with the same clinical signs, but some goats exhibited symptoms of respiratory diseases. The RT-qPCR results show that 58 out of all of the goats were positive for ENTV-2, with a positivity rate of 10.4%. Most of the goats that tested positive for ENTV-2 were 11 months old or 2 years old (Table 2). The positive rate for the male goats was 12.5%, while the positive rate for the female goats was 9.7%. The positive samples (*n* = 58) were detected by using the developed RT-qPCR assay verified in work described previously [8], and a concordance rate of 100% was obtained between them.

**Figure 5:**
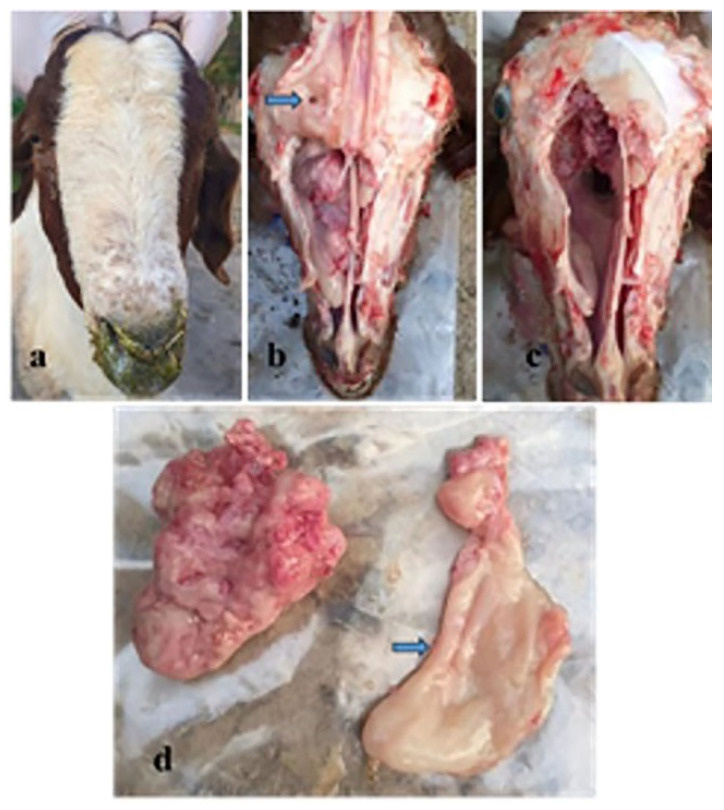
Clinical and gross examination of ENTV-2-affected goats. (**a**) Viscous purulent nasal fluid, facial enlargement, and exophthalmia of a goat infected with ENTV. (**b**,**c**) Unilateral mass in the cranial nasal cavity and perforation of the bone (arrow). (**d**) The tumors were removed from the nasal cavities of the goats with ENA. Softened bone (arrow).

In fact, 3 of the 58 goats showed the typical clinical symptoms of ENA, and these 3 were selected and necropsied. The goats infected with ENTV-2 had viscous purulent nasal fluid, as well as facial enlargement and exophthalmia (Figure 5a). The affected goats were euthanized, and a complete necropsy was performed. The gross examination revealed a tumor occluding the right nasal cavity closely attached to the mucous membrane of the ethmoid turbinates, which infiltrated the frontal and nasal bones (Figure 5b,c). The shape of the mass was irregular, pink, and without hemorrhage, and the mass was firm, measuring approximately 5 cm in diameter (Figure 5d). The tumor undergoes invasive growth into surrounding tissues, resulting in complete softening and perforation of the bone (Figure 5b,d). In all patients, the tumors were restricted to the nasal cavity, and no metastases were detected. In addition, we used the same method to determine the potential virus concentrations in the nasal swabs, tumors, lymph nodes, tracheae, hearts, livers, spleens, lungs, kidneys, and blood of three goats. RT-qPCR analysis revealed that all of the samples were positive for ENTV-2, except for the kidney and negative control samples (Table 3). Overall, the viral load of ENTV-2 was relatively high in the nasal swabs and tumors.

### 3.3. Pathologic and Electron Microscopic Findings of the ENA

**Figure 6:**
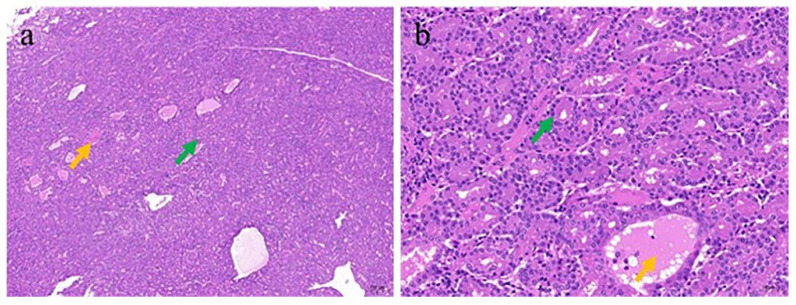
Histopathology of nasal tumors from infected goats. (**a**) The tumor tissue exhibited histological heterogeneity and appeared glandular with a relatively dense arrangement, and it was mostly composed of serous glands (green arrow). Secretions and a rather small amount of shed epithelial cells could be seen within individual glandular lumens (yellow arrow) ((×40) 200 μm). (**b**) The tumor cells were arranged in a glandular pattern, with short columnar shapes and consistent sizes. The lesions were well differentiated (green arrow), with visible secretions inside the cavity (yellow arrow) ((×200) 50 μm).

After H&E staining, the tumor tissue exhibited histological heterogeneity and a relatively dense glandular arrangement, and it was mostly composed of serous glands. Secretions and a rather small amount of desquamated epithelial cells were observed in some glandular lumens (Figure 6a). The neoplastic cells were cuboidal or columnar, generally uniform in size, and exhibited good differentiation. The degree of cellular atypia was minimal, with no prominent nuclear division figures visible (Figure 6b). In addition, lymphocytic infiltration into the interstitia was observed.

**Figure 7:**
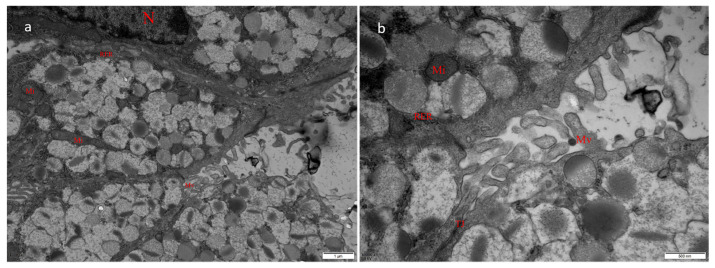
Transmission electron micrograph analysis of ENA tumors. (**a**) Morphological structure of mucus cells in the glandular cavity of nasal proliferative tissue, showing the cell nucleus (N), mitochondria (Mi), rough endoplasmic reticulum (RER), and microvilli (Mv) ((×15,000) 1 μm). (**b**) Enlarged local image of a suspected viral structure (yellow arrow) appearing in the glandular cavity of nasal proliferative tissue, showing mitochondria (Mi), rough endoplasmic reticulum (RER), microvilli (Mv), and tight junctions (TJs), along with mucous particles (blue arrow) ((×40,000) 800 μm).

An electron microscopic observation of the tumors from infected goats (Figure 7a,b) revealed virus particles of similar sizes and morphologies in the glandular cavity. The particles were spherical, and their diameter was approximately 100 nm. The nucleus was elliptical, with a large amount of heterochromatin attached to the nuclear membrane, which was continuous and intact. In the cytoplasm, the mitochondria were elliptical or rod-shaped, with clear, neatly and closely arranged crista structures. The matrix was uniform and showed a homogeneous gray-black color, and the outer membrane of the mitochondria was continuous and intact. The rough endoplasmic reticulum showed a double-layered membrane with narrow lumen spaces, and ribosome particles were attached to the cytoplasmic surface. A large number of mucus granules were distributed in the cytoplasm. Tight junctions could also be seen between the mucus cells, and a small number of sparsely arranged, stubby microvilli were distributed on the free surface.

### 3.4. Complete Genome Characterization of ENTV-2 and Phylogenetic Analysis

Using specific primers, the complete genome sequences of the virus were obtained from three tumor tissues. Sanger dideoxy sequencing revealed that the full-length genomes of two isolates, ENTV-2 SX1 and ENTV-2 SX2, were 7469 nt, while the full-length genome length of ENTV-2 SX3 was 7470 nt. The nucleotide sequence identities from three different isolates were over 98%. A BLAST analysis of the complete genomes of the NCBI revealed that these three isolates had the highest nucleotide identity with ENTV-2FJ (accession number: MK559457.1) and ENTV-2 Shaanxi (accession number: KU179192.1). The coverages with ENTV-2FJ (accession number: MK559457.1), ENTV-2 Shaanxi (accession number: KU179192.1), and ENTV-2FJ (accession number) were 100%, 97%, and 100%, with identities of 97.87%, 97.90%, and 98.53%, respectively. The open reading frames of the gag, pro, pol, and env genes were 1839 nt (nt 258–2096), 927 nt (nt 1931–2857), 2625 nt (nt 2824–5448), and 1869 nt (nt 5324–7192), and they encoded proteins of 612 aa, 308 aa, 870 aa, and 617 aa, respectively.

For the gag protein, the three strains shared greater than 99.3% aa identity, and there were four amino acid mutations, while they showed the highest aa identity with the ENTV-2 reference strains JY (accession number: MT254064.1) and Shaanxi (accession number: KU179192.1) (99.35%, 99.35%, and 99.67%, respectively). The pro-protein sequence of the ENTV-2 SX1~3 strains had high amino acid identities with each other (greater than 96.8%). There were 11 amino acid differences among the pro regions of ENTV-2 SX1~3, 8 of which were located in ENTV-2 SX3, 2 of which were located in ENTV-2 SX2, and only 1 of which was located in ENTV-2 SX1. The pro proteins of strains ENTV-2 SX1, ENTV-2 SX2, and ENTV-2 SX3 had the highest amino acid identities (97.73%, 98.05%, and 99.68%, respectively) with the ENTV-2 strains Shaanxi4 (accession number: KU980912.1) and JY (accession number: MT254064.1). The amino acid sequence of ENTV-2 SX1~3 pol was more than 94.4% identical. The pol protein of the ENTV-2 SX strain had the highest amino acid identity with that of the reference strains JY (accession number: MT254064.1) and Shaanxi2 (accession number: KU980910.1) (98.63%, 98.17%, and 98.74%, respectively). A comparison of 1869 nt (5324–7192) revealed a high similarity rate of over 99.2% among the ENTV-2 SX1~3 *env* genes. There were only seven amino acid differences among the env proteins of ENTV-2 SX1~3, and the similarity was greater than 99%. The amino acid sequences had the highest identity with Shaanxi (accession number: KU179192.1) (99.68%, 99.52%, and 99.52%, respectively).

**Figure 8:**
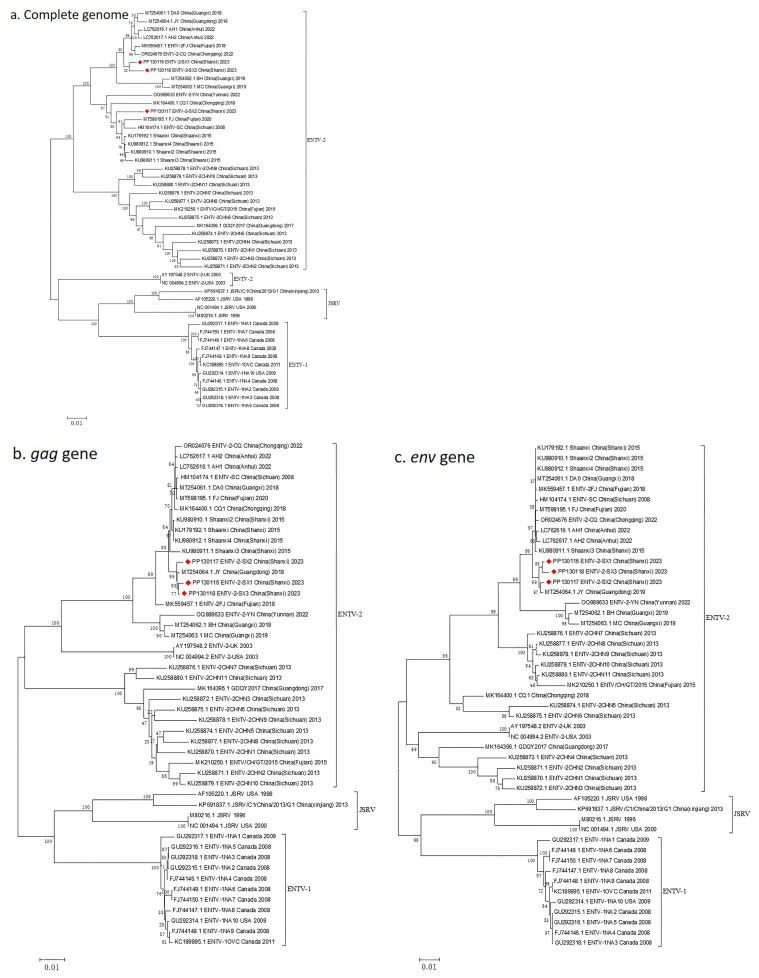
Phylogenetic analysis of caprine and ovine betaretroviruses. Phylogenetic analysis based on the (**a**) complete genome sequence, as well as the (**b**) *gag* and (**c**) *env* genes of strains ENTV-2 SX1~3 and previously published ENTV-2, ENTV-1, and JSRV sequences. The ENTV-2 SX strain described in this study is marked with a solid red diamond.

Multiple alignments based on the complete genome sequences of ENTV-2 showed that ENTV-2 SX1~3 shared high similarity with other Chinese ENTV-2 strains at the nucleotide sequence level. To understand the genetic evolution of ENTV-2 SX1~3, we employed neighbor-joining trees for phylogenetic analysis. The phylogenetic tree based on the complete gene sequences showed that ENTV-2 SX1~3 clustered together with multiple ENTV-2 strains isolated in China during 2015–2023 (Figure 8a). The *env* gene phylogenetic tree was similar to that of *gag*, and the sequences of ENTV-2 SX1~3 were also in a large cluster, with a closer relationship to the JY reference strain (Figure 8b,c). Additionally, ENTV-2 SX1~3 is phylogenetically related to isolates derived from China in recent years, and it is typically geographically clustered.

**Figure 9:**
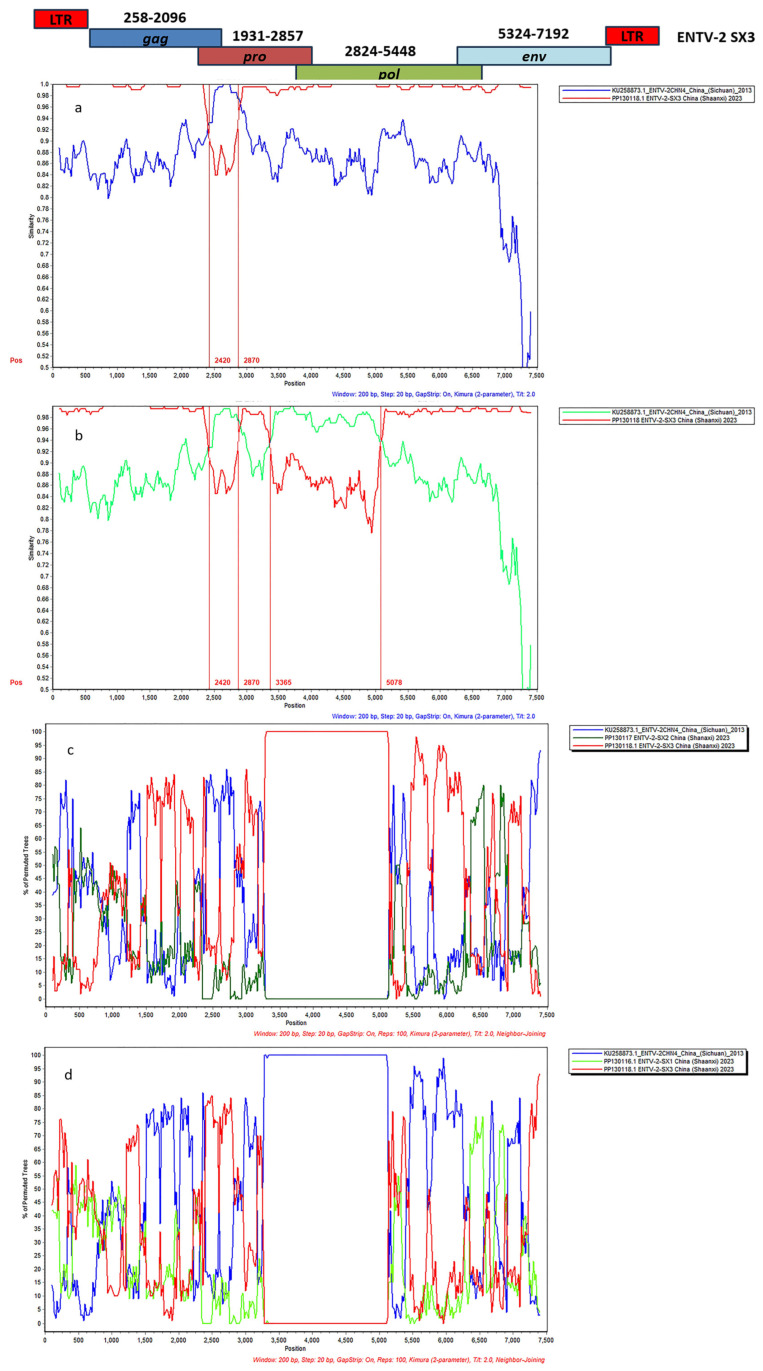
Recombination analysis of the ENTV-2 SX1~3 strains. (**a**) Genome-scale similarity comparisons of strain ENTV-2 SX1 against strains ENTV-2 SX3 and ENTV-2CHN4. (**b**) Genome-scale similarity comparisons of strain ENTV-2 SX2 against strains ENTV-2 SX3 and ENTV-2CHN4. (**c**) A BootScan analysis in the SimPlot software was performed with ENTV-2 SX1 as the query sequence, ENTV-2 SX3 and ENTV-2CHN4 as putative parental isolates, and ENTV-2 SX2 as the outgroup isolate. (**d**) BootScan analysis in the SimPlot software was performed with ENTV-2 SX2 as the query sequence, ENTV-2 SX3 and ENTV-2CHN4 as putative parental isolates, and ENTV-2 SX1 as the outgroup isolate.

In order to validate the possible recombination events and breakpoint positions in the ENTV-2 SX1~3 strains, the full-length genomic sequences of the strains in this study and their 31 potential parental sequences were analyzed using RDP v4.10 and SimPlot v3.5.1 software (Table S1). Recombinant analysis has shown that recombination is widely present in the ENTV-2 genome, and ENTV-2 SX1 and ENTV-2 SX2 might be natural recombinants, with ENTV-2 SX3 as the major parent and ENTV-2CHN4 as the minor parent. Through analysis of the similarity and BootScan plots created with SimPlot v3.5.1 software, the recombination event was observed between the major parent strain ENTV-2 SX3 and the minor parent strain ENTV-2CHN4, with the recombinant region at nucleotides 2420–2870, giving rise to one recombinant strain, ENTV-2 SX1 (Figure 9a,c). Four distinct recombination breakpoints at positions 2420 nt, 2870 nt, 3365 nt, and 5078 nt gave rise to one recombinant strain ENTV-1 SX2(Figure 9b,d). The recombination region consisted of the 3′-terminal *pro* gene at 438 nt and the partial *pol* gene at 2255 nt. The analysis of the viral genome sequence revealed no gene recombination in ENTV-2 SX3.

## 4. Discussion

At present, ENA in goats is prevalent all over the world, and ENTV-2 in China is becoming more prevalent than ever before, causing great economic losses to the goat industry in China. ENTV-2 induces prolonged latent infection, and clinical signs take a while to become obvious, making it difficult to identify ENTV-2-infected animals during the pre-clinical period. However, the absence of a specific humoral immune response following ENTV-2 infection [7] poses challenges related to its serological diagnosis. There is an urgent need to offer a wide array of methods for the detection of ENTV-2. Currently, the diagnosis of ENTV-2 infection relies on a combination of clinical and pathological assessments coupled with PCR detection [1,22]. However, the close genetic relationship among ENTV-2, ENTV-1, JSRV, and EBRVs can potentially compromise the diagnostic specificity of PCR-based techniques used to detect ENTV-2 infections [23]. In the present study, a probe-based RT-qPCR assay was developed for the detection and quantification of ENTV-2 RNA in naturally infected goats. Moreover, modification of the method for use in a qPCR assay enables the detection of proviral DNA in tumor specimens. To the best of our knowledge, this is the first study to describe a real-time PCR assay for detection of the ENTV-2 *pro* gene.

In fact, conventional RT-PCR has defects in its throughput. The TaqMan real-time PCR assay has many advantages over other diagnostic methods. For example, TaqMan probes are preferred over fluorescent dyes because of the improved specificity and precision in the former [24]. Real-time PCR instrumentation requires considerably less hands-on time, and testing is much simpler to perform than conventional PCR methods. Indeed, traditional PCR assays often entail laborious, time-intensive processes and involve an inherent risk of false-positive outcomes [25]. The selection of a suitable target gene for the accurate determination of an animal’s infection status is crucial for the development of real-time PCR for pathogens. In the present study, the real-time assay primers were designed on the basis of the *pro* gene. In addition, the RT-qPCR method that we established not only has higher sensitivity and specificity but also can react in a single reaction tube. More specifically, it has the advantage of the step involving conversion of the RNA to cDNA and the amplification step being performed in the same tube, minimizing cross-contamination and preventing RNA degradation. ENTV-2 is a member of the family *Retroviridae*, which replicate as other retroviruses and in which the RNA genome in the viral particles is reverse-transcribed into a DNA form, the provirus, in infected cells. Therefore, it is possible to modify the method for use in a qPCR assay to enable the detection of pre-viral DNA in tumor samples using the same primers and probes. The detection methods we established exhibited high specificity to ENTV-2, as no amplification was seen when tested against some other common goat respiratory pathogens and EBRVs. The detection limits for viral RNA and proviral DNA were 2.73 × 10^0^ copies and 7.28 × 10^0^ copies, respectively. The amplification curves of both methods showed good correlations between Ct values and template concentrations, and the standard curves showed high linearity and sensitivity. Moreover, to further evaluate the sensitivity of our real-time PCR-based methods, we also compared them to conventional PCR using the same primers. Both methods were 1000 times more sensitive than conventional RT-PCR and were more efficient for ENTV-2 diagnosis, especially when used on ENTV-2 samples containing low numbers of viral copies. Compared with previous two-step fluorescent dye-based real-time RT-PCR techniques [26,27], we reported here a TaqMan-based one-step RT-qPCR assay. Apostolidi et al. [8] reported a probe-based RT-qPCR technique with a limit of detection of 7 × 10^1^ RNA copies/reaction, and thus the minimum detection limit found in this study was lower than theirs. Furthermore, unlike previous studies that used the *gag* and *env* genes and U3 regions, the target gene in our study was *pro.* The gene alignment results showed that the pro gene in this region was relatively conserved and distinct from the gene sequences of ENTV-1, JSRV, and EBRVs; it met the primer design requirements based on software analysis. Moreover, we detected clinical samples in goats from three farms located in Shaanxi Province, China to determine the ENTV-2 infection status in goat herds, and the results show that 58 goats were positive for ENTV-2, indicating a positivity rate of 10.4%. In affected flocks, the prevalence of the disease is typically 0.5–2%, but occasionally, it can be as high as 15% [11]. Moreover, compared with the method described previously [8], the detection results of 58 positive samples indicate a high consistency in ENTV-2-positive diagnosis for our method, demonstrating the high sensitivity, speed, and accuracy of this real-time RT-PCR assay. The typical clinical signs (including copious seromucous nasal discharge, ocular protrusion, and skull deformations) and tumors were observed in the infected goats in the present study. Due to the transformation of cell types and the space-occupying nature of tumors, affected animals exhibit facial asymmetry. At necropsy, in all patients, a neoplastic mass was observed within the nasal cavity; interestingly, all of the masses were unilateral. In this study, the clinicopathological manifestation of the disease was similar to those described previously. For ENA, many case reports have shown histopathological tumor images. According to the H&E staining results, the tumor was considered to be a nasal adenoma, which is consistent with the features of ENA. It is worth noting that we observed the tumor undergoing invasive growth into surrounding tissues, resulting in complete softening and perforation of the bone. Although ENA is a low-grade adenocarcinoma with well-differentiated vasculature and few nuclear division figures in the classification, the bone destruction suggests that ENA may have malignant potential. To verify that ENTV-2 was present in the nasal tumors of the infected goats, ENTV-2-like particles were observed via TEM, which further provided evidence for tumor formation via ENTV-22 infection in goats. Electron microscopic examination confirmed the glandular character of the neoplasia, which is consistent with previous reports [6]. Histological and ultrastructural studies have suggested that the serous glands of the nasal mucosa are the probable origin of the neoplastic cells. Normally, the tumor latency is between 12 and 18 months, and the disease occurs more frequently in adult animals aged between 2 and 4 years [6,28]. In our study, most of the goats that tested positive for ENTV-2 were 11 months old or 2 years old, and most of the goats in this study were male. Importantly, previous studies have shown that younger goats, such as those which are 3 months old [14] and 6 months old [29], may also be infected with ENTV-2. Therefore, the possibility of transuterine and oral transmission cannot be excluded or ruled out. To date, neither breed nor sex or genetic predisposition has been reported in any published study [2]. Previous studies have suggested that the prevalence of ENA is quite similar in different breeds of sheep and in both sexes [30,31]. Our research results show that the positivity rate of male goats (18/144, 12.5%) was higher than that of female goats (40/414, 9.7%). This indicates that male goats may be more susceptible to ENTV-2. ENTV-2 is similar to ENTV-1, but they may have significantly different biological characteristics. It is thus necessary to further study the prevalence of ENA in different breeds of goats and both sexes, which may contribute to the understanding of ENA.

In our study, ENTV-2 was detected mainly in the tumor, nasal fluid, and tracheal tissue samples. It was not detected in the kidney tissues of the three goats, which is consistent with the findings of previous research [1]. The virus content in the blood is relatively low. Therefore, the best samples for detecting this virus in epidemiological investigations comprise nasal fluid. In addition, three full-length ENTV-2 sequences were obtained from three goat flocks in Shaanxi Province, China. Multiple sequence alignment showed that the similarity rates among ENTV-2 SX1-3 are extremely high at both the nucleotide and amino acid levels, and the similarity rates with other strains from China are higher than those of foreign strains. On the other hand, most of the variations in ENTV-2 SX1-3 are conserved, indicating that the three isolates obtained in this study have unique characteristics distinct from other ENTV-2 strains. Phylogenetic analysis through nucleotide sequences showed that the three ENTV-2 isolates of Shaanxi Province were related to the Chinese ENTV-2 isolates and were typically geographically clustered. This finding indicates that the rapid expansion of the logistics industry, which has facilitated the increasingly frequent movement of live goats and mutton products, has also accelerated the spread of this infectious virus from other areas in China.

To further investigate the origin of the gene variation in ENTV-2 SX1~3, a recombination analysis based on the complete genome sequences of ENTV-2 was conducted. It was found that recombination is widely present in the ENTV-2 genome. Importantly, three potential intra-genotypic recombination events between strains ENTV-2 SX3 and ENTV-2CHN4 were found. Furthermore, it was indicated that ENTV-2 SX3 from Shaanxi Province could be the major parental strain of both ENTV-2 SX1 and ENTV-2 SX2, and ENTV-2CHN4 from Sichuan Province could be the minor parental strain. The recombination events principally occurred in the *pro* and *pol* gene regions of ENTV-2 in this study, which is consistent with previous findings [32]. The *pro* gene mainly encodes two proteins; one is a protease with deoxyuridine triphosphate activity, and the other is an active protease (PR). The *pol* gene encodes the basic retroviral enzymes of the reverse transcriptase (RT), the RNase H, and the integrase [33]. These proteins are all related to virus replication. Future studies are needed to determine whether the recombination occurring in the *pro* and *pol* gene regions has an impact on the viral and biological characteristics and pathogenesis. Through this study, we have not only gained insights into the evolutionary relationships of the newly emerged ENTV-2 strains in Shaanxi but also found that ENTV-2 SX1 and ENTV-2 SX2 may be previously unreported recombinant strains. The occurrence of the recombination events is related to the presence of ENTV-2 SX3 in this region. The movement of live animals, contaminated fomites, and human activities across diverse farms may have led to cross-infection of ENTV-2 in the goat population, leading to the emergence of new variants. Whole-genome characterizations of newly emerging Shaanxi ENTV-2 field strains will provide more detailed insights into ENTV-2 mutations and recombination, as well as their relationships with molecular epidemiological features.

In summary, real-time PCR-based methods were established here for the detection of ENTV-2, and they were found to be suitable for the diagnosis of ENTV-2 infection and epidemiological surveillance. Moreover, we performed an ENTV-2 infection survey with three flocks from Shaanxi Province and obtained three full-length sequences. Each sequence was determined to be unique, and the three isolates had unique characteristics. Phylogenetic analysis indicated that all of the ENTV-2 SX isolates were related to the Chinese ENTV-2 isolates and showed the highest homology with the Chinese JY isolate (accession number: MT254054.1). Sequence analysis suggests that ENTV-2 SX1 and ENTV-2 SX2 may be recombinant strains associated with the cross-infection of ENTV-2 in the local area. The findings in this study will contribute to a better understanding of the transmission and evolution of ENTV-2, providing new insight into the evolutionary patterns of the virus.

## Figures and Tables

**Table 1:** The sensitivity and repeatability of the established ENTV-2 RT-qPCR and qPCR assays.

Type	Copy Number	Ct (Mean ± S.D.)	CV%	Type	Copy Number	Ct (Mean ± S.D.)	CV%
Standard RNA ^a^	2.73 × 10^8^	8.986 ± 0.058	0.946	Standard DNA ^b^	7.28 × 10^8^	9.813 ± 0.184	1.875
	2.73 × 10^7^	12.208 ± 0.012	0.098		7.28 × 10^7^	13.212 ± 0.147	1.113
	2.73 × 10^6^	15.467 ± 0.042	0.268		7.28 × 10^6^	15.989 ± 0.143	0.894
	2.73 × 10^5^	18.904 ± 0.312	1.650		7.28 × 10^5^	19.478 ± 0.069	0.354
	2.73 × 10^4^	22.288 ± 0.081	0.363		7.28 × 10^4^	22.587 ± 0.118	0.522
	2.73 × 10^3^	25.398 ± 0.950	3.740		7.28 × 10^3^	25.503 ± 0.229	0.898
	2.73 × 10^2^	29.224 ± 0.281	0.962		7.28 × 10^2^	28.862 ± 0.131	0.454
	2.73 × 10^1^	32.896 ± 0.110	0.334		7.28 × 10^1^	32.877 ± 0.193	0.587
	2.73 × 10^0^	36.023 ± 0.450	1.249		7.28 × 10^0^	36.855 ± 0.949	2.575
	NC	None	None		NC	None	None

^a^ Standard RNA was used as a template for the detection of ENTV-2 viral RNA. ^b^ Standard DNA was used as a template for the detection of ENTV-2 proviral DNA.

**Table 2:** Sample information of goats taken for detection by RT-qPCR.

Age	Sex	Number of Samples	Number of Positive Samples (%)
8 years old	♀	1	0 (0%)
7 years old	♀	11	0 (0%)
6 years old	♂	1	0 (0%)
	♀	29	1 (3.4%)
5 years old	♂	1	0 (0%)
	♀	38	1 (2.6%)
4 years old	♂	4	0 (0%)
	♀	18	0 (0%)
3 years old	♂	7	0 (0%)
	♀	57	7 (12.3%)
2 years old	♂	24	1 (4.2%)
	♀	91	17 (18.5%)
1 year old	♂	14	0 (0%)
	♀	36	0 (0%)
11 months old	♂	93	17 (18.3%)
	♀	133	14 (10.5%)

♂ indicates male; ♀ indicates female.

**Table 3:** The number of ENTV-2 RNA copies/μL of template in the samples from the three goats, tested via specific real-time RT-PCR.

Sample	Goat 1	Goat 2	Goat 3
Nasal swab	3.82 × 10^5^	1.57 × 10^5^	1.08 × 10^5^
Tumor	2.26 × 10^7^	7.34 × 10^7^	1.90 × 10^7^
Lymph node	1.76 × 10^1^	2.93 × 10^2^	2.96 × 10^1^
Trachea	4.94 × 10^4^	4.87 × 10^4^	4.77 × 10^4^
Heart	4.59 × 10^1^	1.01 × 10^2^	9.34 × 10^1^
Liver	9.86 × 10^1^	2.15 × 10^2^	1.16 × 10^2^
Spleen	2.76 × 10^1^	4.72 × 10^1^	5.08 × 10^1^
Lung	5.98 × 10^1^	4.43 × 10^2^	5.26 × 10^1^
Kidney	None	None	None
Blood	9.11 × 10^0^	1.46 × 10^1^	2.46 × 10^1^

## Data Availability

The data in this study are available from the authors upon reasonable request. The complete genome sequences of ENTV-2 SX1, ENTV-2 SX2, and ENTV-2 SX3 have been submitted to the GenBank database with accession numbers PP130116.1, PP130117.1, and PP130118.1, respectively.

## Supplementary Materials

The following supporting information can be downloaded at https://www.mdpi.com/article/10.3390/genes16050529/s1. Table S1. Information on the 31 ENTV-2 strains from GenBank for recombination analysis.
