# Supplementary material for: Development and Application of a TaqMan-Based qPCR Assay for Detecting ENTV-2 in Goats
Source: Genes (Basel). 2025 Apr 29;16(5):529. doi: 10.3390/genes16050529 (PMC12111429; doi:10.3390/genes16050529)
Supplement: Supplementary file 1 [file genes-16-00529-s001.zip › genes-3575665-supplementary.pdf]

Table S1. Information of the 31 ENTV-2 strains from GenBank for recombination analysis.

Accession number	Name	Country/region	Year
MK559457.1	ENTV-2FJ	China, Fujian	2018
OR965522.1	ENTV-2 JX	China, Jiangxi	2023
LC762616.1	ENTV-2 AH1	China, Anhui	2022
LC762617.1	ENTV-2 AH2	China, Anhui	2022
OR682176.1	CQ2	China, Chongqing	2022
PP682590.1	ENTV-2 YN2023	China, Yunnan	2023
OR024676.1	ENTV-2-CQ	China, Chongqing	2022
OQ989633.2	ENTV-2-YN	China, Yunnan	2022
MK210250.1	ENTV/CH/GT/2015	China, Fujian	2015
HM104174.1	ENTV-SC	China, Sichuan	2008
KU179192.1	Shaanxi	China, Shaanxi	2015
KU980910.1	Shaanxi2	China, Shaanxi	2015
KU980911.1	Shaanxi3	China, Shaanxi	2015
KU980912.1	Shaanxi4	China, Shaanxi	2015
KU258870.1	ENTV-2CHN1	China, Sichuan	2013
KU258871.1	ENTV-2CHN2	China, Sichuan	2013
KU258872.1	ENTV-2CHN3	China, Sichuan	2013
KU258873.1	ENTV-2CHN4	China, Sichuan	2013
KU258875.1	ENTV-2CHN6	China, Sichuan	2013
KU258876.1	ENTV-2CHN7	China, Sichuan	2013
KU258877.1	ENTV-2CHN8	China, Sichuan	2013
KU258878.1	ENTV-2CHN9	China, Sichuan	2013
KU258879.1	ENTV-2CHN10	China, Sichuan	2013
KU258880.1	ENTV-2CHN11	China, Sichuan	2013
MK164396.1	GDQY2017	China, Guangdong	2017
MK164400.1	CQ1	China, Chongqing	2018
MT254061.1	DA0	China, Guangxi	2018
MT254062.1	BH	China, Guangxi	2019
MT254063.1	MC	China, Guangxi	2019
MT598195.1	FJ	China, Fujian	2020
PQ178888.1	ENTV-2FJ2024	China, Fujian	2024
